# Fast ortho-to-para conversion of molecular hydrogen in chemisorption and matrix-isolation systems

**DOI:** 10.3389/fchem.2023.1258035

**Published:** 2023-08-29

**Authors:** Hirokazu Ueta, Katsuyuki Fukutani, Koichiro Yamakawa

**Affiliations:** ^1^ Advanced Science Research Center, Japan Atomic Energy Agency, Ibaraki, Japan; ^2^ Institute of Industrial Science, The University of Tokyo, Tokyo, Japan

**Keywords:** hydrogen, nuclear spin, rotational energy, adsorption, surface, matrix isolation

## Abstract

Molecular hydrogen has two nuclear-spin modifications called *ortho* and *para*. Because of the symmetry restriction with respect to permutation of the two protons, the *ortho* and *para* isomers take only odd and even values of the rotational quantum number, respectively. The *ortho*-to-*para* conversion is promoted in condensed systems, to which the excess rotational energy and spin angular momentum are transferred. We review recent studies on fast *ortho*-to-*para* conversion of hydrogen in molecular chemisorption and matrix isolation systems, discussing the conversion mechanism as well as rotational-relaxation pathways.

## 1 Introduction

Molecular hydrogen has a remarkable feature of nuclear-spin isomers. According to the quantum number *I* of the total nuclear spin, a hydrogen molecule has two modifications called *ortho* (*I* = 1) and *para* (*I* = 0) species. Because of the symmetry restriction, *ortho* (*para*) H_2_ takes only odd (even) values of the rotational quantum number *J*. Whereas the transition between *ortho* and *para* species is strictly forbidden in the isolated state, it is significantly promoted upon interaction with other substances. Because the nuclear-spin isomers are identified by *I* and *J*, the *ortho*-to-*para* (*o-p*) conversion of H_2_ involves two aspects of the nuclear-spin flip and the rotational-energy transfer to a surface or a surrounding material.

The gas-surface energy transfer has been one of the main topics in surface physics and chemistry; a typical example is the adsorption event, where the translational and adsorption energy of a molecule is transferred to a surface. In addition to the translational energy, internal degrees of freedom such as molecular vibration and rotation are also of considerable importance. Owing to the advance in laser techniques, the relaxation processes of vibrationally-excited molecules have been well studied to date (Beckerle et al., 1990; Chang and Ewing, 1990; Morin et al., 1992; Laß et al., 2005; Chen et al., 2019; Kumar et al., 2019). Compared to the vibration, on the other hand, the energy transfer from the molecular rotation to surface degrees of freedom has been poorly understood. One promising approach for the elucidation of the rotational-energy transfer is to investigate the *o-p* conversion of H_2_ on surfaces and in matrices. After adsorption on a cold surface or trapping in a matrix, *ortho* (*para*) H_2_ predominantly occupies its lowest rotational level with *J* = 1 (*J* = 0). Upon *o-p* conversion of H_2_, the rotational-energy needs to be dissipated into surface or matrix degrees of freedom.

The *o-p* conversion of H_2_ has been observed on various systems (Fukutani and Sugimoto, 2013). The *o-p* conversion is closely related to the quest for efficient ways of the H_2_ storage (Ilisca, 2021), and therefore is being investigated not only on surfaces but also inside solids (Lavrov and Weber, 2002; Hiller et al., 2007; Peng et al., 2009) and nano-cages (Carravetta et al., 2004; Carravetta et al., 2006; Carravetta et al., 2007). Stimulated by the development of the experimental investigations, several conversion mechanisms have been also proposed depending on the interacting materials (Yamakawa and Fukutani, 2020): the Wigner model (Wigner, 1933), where the proton spin interacts with the inhomogeneous magnetic field generated by localized paramagnetic ions; the electron-spin-induced conversion models categorized into the second- (Ilisca, 1991) and third-order (Ilisca and Ghiglieno, 2016) perturbation theories, where the virtual electron exchange or transfer between H_2_ and a surface is involved along with the Fermi contact interaction between an electron and a proton in H_2_; the electric-field-induced conversion model (Sugimoto and Fukutani, 2011), where the Stark, spin-orbit, and Fermi-contact couplings mix the *ortho* and *para* states.

In spite of the extensive studies on the H_2_ *o-p* conversion in the last decades, there still remain controversial issues; one is fast conversion and the other is the rotational-energy dissipation. In contrast to the *o-p* conversion time of  ~ 10^3^ s or longer observed on various surfaces and in solids, H_2_ *o-p* conversion with a time constant shorter than ~ 10^2^ s was recently observed on Pd(210) and inside a molecular solid of CO_2_. On the Pd(210) surface, furthermore, rotational-energy transfer was investigated in detail taking account of electrons and phonons of surfaces. In this review paper, we expound the studies of the fast *o-p* conversion in a CO_2_ matrix (Section 2) and on Pd(210) (Section 3), discussing the spin and rotational-energy transfer.

## 2 Matrix isolation system

The techniques of nuclear magnetic resonance (NMR), neutron scattering, Raman spectroscopy, and infrared absorption spectroscopy have been applied to *in situ* observation of the H_2_ conversion in fullerene (C_60_) (Carravetta et al., 2004; 2006; 2007), metal-organic frameworks (MOFs) (FitzGerald et al., 2010), porous coordination polymers (Kosone et al., 2015), semiconductors (Lavrov and Weber, 2002; Hiller et al., 2007; Peng et al., 2009), and viscous organic solutions (Aroulanda et al., 2007), as was reviewed recently (Ilisca, 2021). Whereas NMR directly probes the nuclear spin, the other methods enable one to resolve the rotational states of *ortho*- and *para*-H_2_. In the nuclear-spin conversion study of polyatomic molecules such as H_2_O (Fajardo et al., 2004; Abouaf-Marguin et al., 2007; Fillion et al., 2012), NH_3_ (Boissel et al., 1993; Gauthier-Roy et al., 1993; Ruzi and Anderson, 2013), and CH_4_ (Miyamoto et al., 2008; Sugimoto et al., 2015; Sugimoto and Yamakawa, 2021), the most popular technique has been rovibrational spectroscopy combined with the matrix-isolation method, where target molecules are isolated in molecular solids, e.g., rare-gas ones. Indeed, the temperature dependence of the conversion rate has been intensively studied in this way to reveal the pathways for the rotational relaxation (Sugimoto et al., 2015; Turgeon et al., 2017; Yamakawa et al., 2017).

Since H_2_ is the lightest molecule and has a relatively small interaction with matrix molecules, rather low temperatures (typically *T* < 15 K) are required to suppress its diffusion and formation of aggregates. In previous studies, the *ortho* and *para* isomers of H_2_ have been separately detected in various matrices of Ar, Kr, Xe, N_2_, and CO by using Raman spectroscopy (Prochaska and Andrews, 1977; Alikhani et al., 1989; Kornath et al., 1999). In particular, Alikhani *et al.* kept Ar-isolated H_2_ at 9 K for 24 h and observed no change of the intensity ratio of the *ortho* and *para* signals, which means the *o-p* conversion was suppressed significantly in solid Ar. In contrast, comparing the ratio with a calculated value, the *o-p* conversion was found to partially proceed just during the sample deposition; they pointed out the possibility that this conversion was catalyzed by O_2_ impurities. Although H_2_ has no permanent electric dipole moment, one is able to detect matrix-isolated H_2_ also by infrared absorption spectroscopy because of its weak polarization. Warren et al. measured infrared spectra of H_2_ trapped inside Ar, Kr, N_2_, and CO matrices in the wavenumber regions of pure rotational and vibrational transitions; except for the rotational spectrum of Kr-isolated H_2_, the *ortho* and *para* signals were resolved (Warren et al., 1980). They also found the *ortho*-to-*para* ratio of H_2_ trapped in solid Ar to be decreased by ∼25% after 2–3 days and attributed this *o-p* conversion to the accidental contamination of magnetic impurities, though the accurate conversion rate was not determined. In a recent study (Yamakawa et al., 2020), H_2_ was trapped and polarized in solid CO_2_, so that the conversion rate of H_2_ was derived from the time evolution of its infrared absorption band, as is expounded below.

**FIGURE 1:**
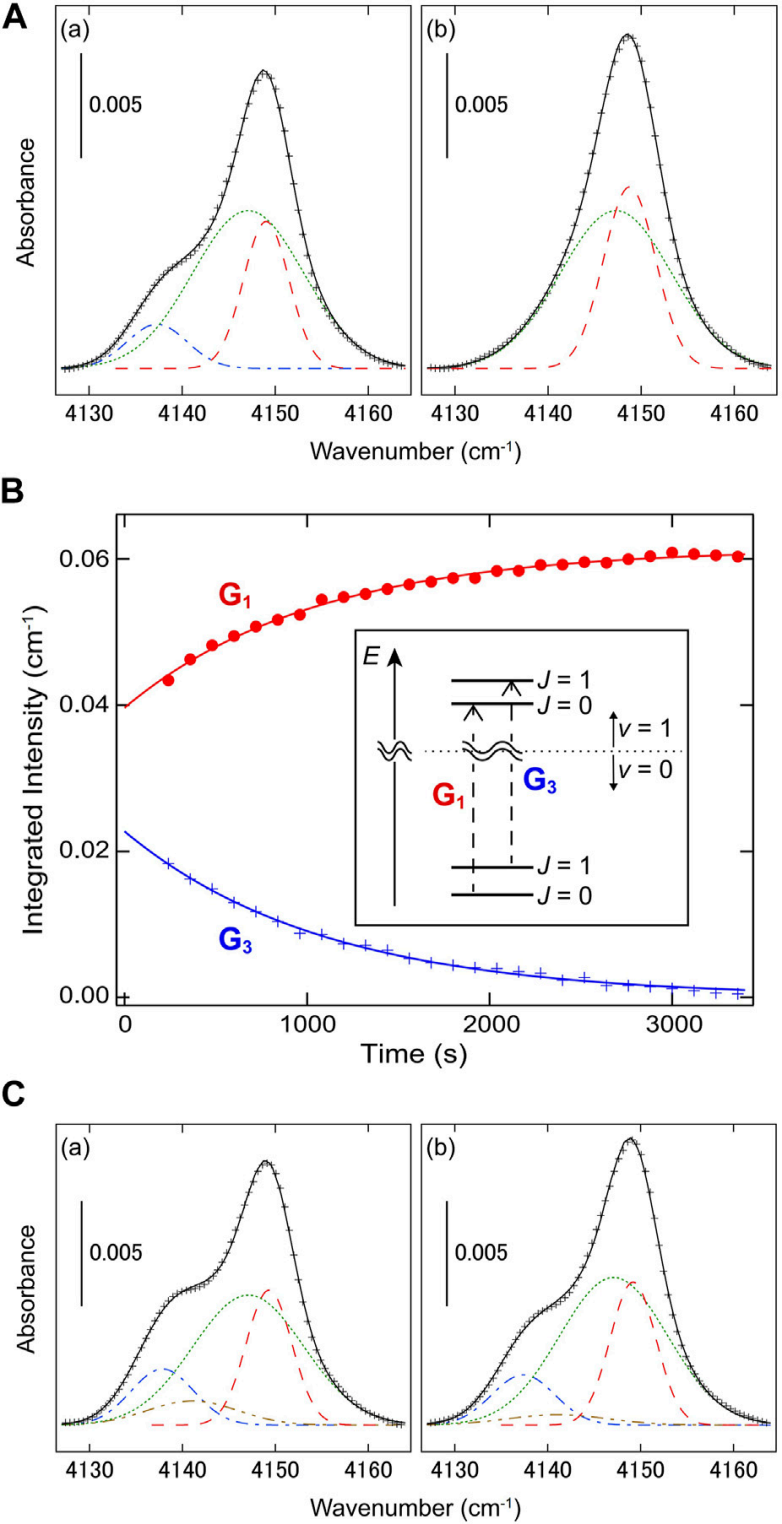
Nuclear spin conversion of H_2_ trapped in solid CO_2_ at 5.4 K (Yamakawa et al., 2020) (Copyright 2020; American Physical Society). **(A)** Induced infrared-absorption bands of H_2_ (a) 240 s and (b) 3,360 s after the sample deposition. The result of multi-gaussian fitting is also shown; the gaussian curves of G_1_, G_2_, and G_3_, are represented by dashed, dotted, and dashed-dotted lines, respectively. **(B)** The integrated intensities of G_1_ and G_3_ are plotted against time. The solid lines denote the result of monoexponential fitting. The vibrational transitions of H_2_ corresponding to G_1_ and G_3_ are schematically shown in the inset. **(C)** Induced infrared-absorption bands of H_2_ (a) 0 s and (b) 120 s after the sample deposition. The result of tetra-gaussian fitting is also shown; G_1_, G_2_, G_3_, and G_4_, are denoted by dashed, dotted, dashed-dotted, and dashed double-dotted lines, respectively.

The room-temperature gaseous mixture of CO_2_ and H_2_ at a molar ratio of CO_2_/H_2_ = 100 was condensed onto a gold substrate at 5.4 K for 10 min, and infrared spectra were measured in the reflection configuration. From the film interference pattern appearing in the baseline of the spectrum, the thickness of the CO_2_ matrix was determined to be 4.5 μm . Just after the condensation, the spectrum showed not only intense absorption bands of CO_2_ but also a weak band of H_2_. At a trapping site of H_2_, the electric fields generated by surrounding CO_2_ molecules did not cancel each other out, resulting in slight electric-polarization of H_2_. The time evolution of the H_2_ band after the sample deposition is displayed in Figure 1A. The absorption band was well-reproduced by the combination of three gaussian curves: G_1_ at 4,149 cm^−1^, G_2_ at 4,147 cm^−1^, and G_3_ at 4,138 cm^−1^. Whereas G_1_ grew with increasing time, G_3_ decayed and finally disappeared. This time development was attributed to the conversion of H_2_ from *ortho* to *para*; in other words, G_1_ and G_3_ were assigned to the Q_1_ (0) and Q_1_ (1) transitions of *para*- and *ortho*-H_2_, respectively. In the gas phase, the transition energy of Q_1_ (0), 4,161.1 cm^-1^, is also higher than that of Q_1_ (1), 4,155.3 cm^−1^ (Dabrowski, 1984), owing to the rovibrational coupling in H_2_. As shown in Figure 1B, the integrated intensities of G_*m*_ (*m* = 1, 3) were analyzed as a function of time, and were found to follow the monoexponential function:

$$I_{m}\left(t\right)=\left[I_{m}\left(0\right)-I_{m}\left(\infty \right)\right]\;\exp\left(-k_{m}t\right)+I_{m}\left(\infty \right),$$

where $I_{m}\left(0\right)$ and $I_{m}\left(\infty \right)$ denotes the initial and equilibrium intensities, respectively. The conversion rate derived from G_1_, $k_{1}=\left(9.6\pm 0.4\right)\times 10^{-4}\;s^{-1}$ , coincided within error with that from G_3_, $k_{3}=\left(9.2\pm 0.4\right)\times 10^{-4}\;s^{-1}$ .

To reveal the origin of G_2_, Yamakawa *et al.* also investigated the time evolution of the infrared spectrum just after the sample deposition, as shown in Figure 1C; in the time range of t=0−120 s , an additional component, G_4_, was detected at 4,141 cm^−1^, and the decay of G_4_ was observed simultaneously with the growth of G_2_. Thus, it was likely that there were two kinds of trapping sites for H_2_ inside solid CO_2_; while G_1_ and G_3_ were attributed to H_2_ at site A, G_2_ (G_4_) was to *para* (*ortho*) H_2_ at site B. The frequency difference between the *para* and *ortho* species at site B was about a half of that at site A. This suggests the approach of the lowest rotational levels of *ortho*- and *para*-H_2_ and relatively high anisotropy of the confining potential at site B. Note that the estimated *o*-*p* conversion rate at site B was as high as $6\times 10^{-3}\;s^{-1}$ . In a previous study with use of electron-energy-loss spectroscopy, the conversion rate of H_2_ adsorbed on the stepped surface of Cu(510), where the adsorption potential is strongly anisotropic, was evaluated to be on the order of 1 s (Svensson and Andersson, 2007). These results suggest fast conversion of rotationally hindered H_2_, which is also shown in Section 3.

In most of the condensed systems, the vibrational-frequency shift of H_2_ with respect to the gas phase, $\Delta Q_{1}\left(0\right)$ , was negative: −12 ( −14 ) cm^-1^ at site A (B) of solid CO_2_ (Yamakawa et al., 2020), −17 cm^-1^ in solid N_2_ and CO (Warren et al., 1980), −20 cm^-1^ on amorphous D_2_O ice (Hixson et al., 1992). Despite the relatively small red-shift, the conversion rate of H_2_ in solid CO_2_ at 5.4 K was even higher than that on amorphous H_2_O ice at 9.2 K, $2.4\times 10^{-4}\;s^{-1}$ , measured by Ueta et al., 2016, who also reported the monotonical increase of the rate with temperature below 14 K. This result is not explained by the electric-field-induced conversion mechanism (Sugimoto and Fukutani, 2011); instead, probable is the three-step conversion model (Ilisca and Ghiglieno, 2016; Ilisca, 2018), which consists of electron exchange between H_2_ and a solvent, hyperfine contact interaction in H_2_, and spin-orbit interaction inside the solvent.

Interestingly enough, the conversion time-constants (the inverse of the conversion rate) of H_2_ in non-magnetic systems accompanied by energy gaps are distributed quite widely: a few or tens of minutes in MOFs (FitzGerald et al., 2010) and solid CO_2_ (Yamakawa et al., 2020), and tens or hundreds of hours in crystalline Si (Lavrov and Weber, 2002; Hiller et al., 2007; Peng et al., 2009) and the cage of C_60_ (Chen et al., 2013). Note that the CO_2_ film deposited below ∼9 K has a porous structure with the enhanced surface area and exhibits a unique feature of “thermal spikes”, which are abrupt temperature rises due to the structural rearrangement during the film deposition (Arakawa et al., 1979). The enhanced *o-p* conversion of H_2_ is possibly related to the characteristically unstable and porous structure of the CO_2_ film. In order to further investigate the origin of the large difference in the conversion time, the electronic structure of the H_2_-solvent system, including the anisotropy of a confining potential, should be also studied. The temperature dependence of the conversion rate of H_2_ trapped in matrices is under investigation and will bring about information on the channels of the rotational relaxation, just like the surface system described in the following section.

## 3 Molecular chemisorption system

As typical adsorption schemes of H_2_, dissociative chemisorption and molecular physisorption are recognized. In most of past studies on *o-p* conversion at surfaces, H_2_ in the physisorption state via the van der Waals interaction was focused, in which the molecule is in a nearly-free rotational state (Stulen, 1988; Sugimoto and Fukutani, 2014). On the other hand, some stepped surfaces exhibit a peculiar adsorption of molecular chemisorption (Mårtensson et al., 1986; Svensson et al., 1999; Schmidt et al., 2001; Sun et al., 2004; Svensson and Andersson, 2007; Christmann, 2009; Shan et al., 2009), in which the adsorption potential is anisotropic with respect to the molecular-axis angle and the rotational motion is strongly hindered. Although the rotational state is modified under the anisotropic potential, the rotational state of H_2_ chemisorbed on surfaces is correlated with the nuclear-spin state, either *ortho* or *para*. In the past, while the occurrence of fast *o-p* conversion under an anisotropic potential was indicated for the systems of Cu(510) and Pd(210), direct evidence was lacking due to the limited time resolution of the experimental technique used in previous studies (Svensson and Andersson, 2007; Ohno et al., 2018). In this section, fast *o-p* conversion and associated rotational-energy transfer are discussed for H_2_ molecularly chemisorbed on Pd(210).

The Pd(210) surface has (100) terraces with steps running along the [001] direction forming open (110)-like microfacets. H_2_ chemisorbs on the step-edge of Pd atoms, so that H_2_ binds strongly to the surface compared with the physisorption systems (Schmidt et al., 2001). The adsorption state of H_2_ has been studied experimentally and theoretically (Lischka and Groß, 2002; Arguelles and Kasai, 2018a; Arguelles and Kasai, 2018b). The H_2_ adsorption potential is highly anisotropic, which induces lifting of the rotational state degeneracy of the triply degenerate *J* = 1 state in gas-phase into doubly degenerate state (*m* = ±1, *m*: *z* component of *J*) and a non-degenerate state (*m* = 0) in the adsorption state. Hence the lowest *o-*H_2_ (*m* = ±1) behaves like a two-dimensional rotor (Svensson et al., 1999).

**FIGURE 2:**
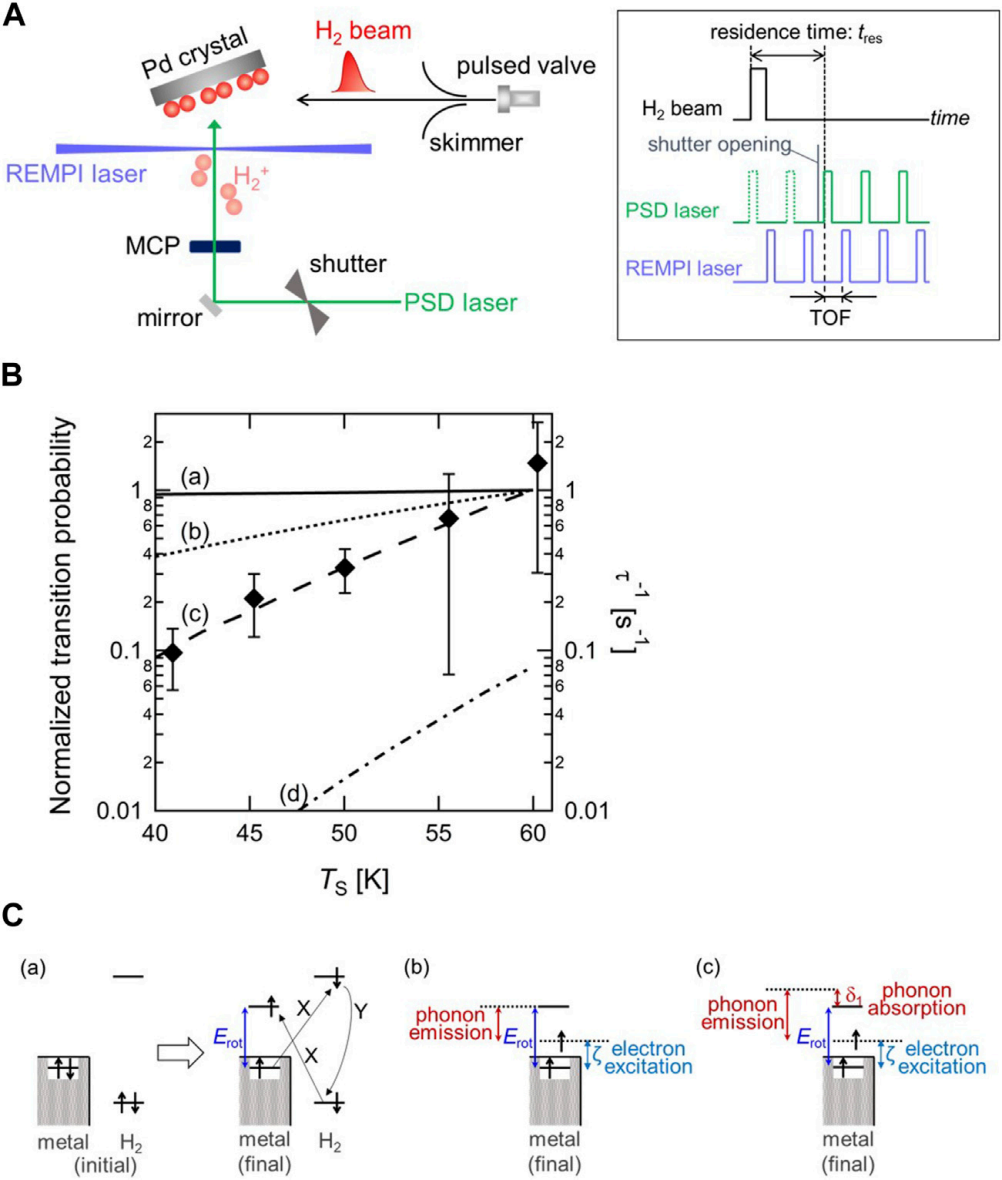
**(A)** Schematic diagram of the experimental setup and pulse sequence driving the molecular beam and two lasers for the *o-p* conversion measurement (Ueta et al., 2020) (Copyright 2020; American Physical Society). **(B)** Surface temperature dependence of the *o-p* conversion rate (τ^−1^) with calculated *o-p* transition probability through (a) the *XY* model (solid line), (b) a combination model of electron transition and one-phonon process (dotted line), and (c) a combination model of electron transition and two-phonon process (dashed line). The value of transition probability at *T*_S_ = 60 K is normalized to 1 for three models. On the other hand, (d) the *p*-*o* transition probability through a combination model of electron transition and two-phonon process at *T*_S_ = 60 K is normalized to the value of the *o*-*p* transition probability through the same model at the same *T*_S_ (dashed-dotted line). Note that the experimentally determined conversion rates correspond to right axis, and the results of model calculation correspond to left axis (Ueta and Fukutani, 2023) (Copyright 2023 American Chemical Society). **(C)** Schematic illustration of the rotational-energy transfer in *o-p* conversion through (a) the *XY* model, (b) the electronic excitation and one-phonon process, and (c) the electronic excitation and two-phonon process. *X* and *Y* denotes the Coulomb interaction and the Fermi contact hyperfine interaction, respectively (Ueta and Fukutani, 2023) (Copyright 2023 American Chemical Society).

To track the fast *o-p* conversion directly, a new experimental method was developed by combining a pulsed molecular beam (MB), photo-stimulated desorption (PSD), and resonance-enhanced multiphoton ionization (REMPI). Figure 2A shows a schematic diagram of the experimental setup and a timing chart of the MB-PSD–REMPI measurement for probing the time evolution of the rotational states of H_2_ on surfaces (Ueta et al., 2020). Probing the change in the rotational state distribution allows us to track *o-p* conversion owing to the fact that the rotational states of H_2_ couple with nuclear spins. With *n-*H_2_ molecular beam deposition, the *o-p* ratio at a surface temporarily becomes out-of-thermal-equilibrium, and then relaxes to the thermal equilibrium. From the change in the two nuclear-spin-state populations as a function of the adsorption time, the conversion rate can be determined, similar to that shown in Figure 1B. The conversion rate has been successfully determined on Pd(210) as a function of the surface temperature (*T*_S_) in the range of 41–60 K as shown in Figure 2B. It is found the conversion rate increases with increasing temperature. Note that the values of the conversion time (inverse of conversion rate) are on the order of 1–10 s, demonstrating the occurrence of fast conversion on Pd(210).

Since the *o-p* conversion is accompanied by the rotational transition as well as the nuclear spin flip, the surface temperature dependence of *o-p* conversion allows us to investigate the rotational-energy (*E*_rot_) transfer process in *o-p* conversion. The rotational-energy dissipation process has been discussed in previous studies on the *o-p* conversion of physisorbed H_2_ on amorphous solid water, silicate and carbon materials (Ueta et al., 2016; Tsuge et al., 2021; Tsuge et al., 2021). These studies suggested that substrate phonons play an important role in the rotational-energy transfer in the conversion. Depending on the substrate, two kinds of phonon dissipation process have been considered in those studies; one-phonon and two-phonon processes. Whereas in the former process the rotational energy is dissipated into the surface by excitation of a phonon, the latter process proceeds via the simultaneous absorption of a phonon from the initial up to an intermediate state, and the emission of another from the intermediate to the final states (Scott and Jeffries, 1962). Since those materials are all non-metallic, the influence of surface electrons in the rotational-energy transfer process can be neglected.

On the other hand, Pd is a non-magnetic metal, where substrate electrons are expected to play an important role in analogy with the vibrational-energy relaxation. A widely accepted conversion model on a non-magnetic metal surface is the electron-exchange-hyperfine-contact (*XY*) model proposed by Ilisca (Ilisca, 1991) (Figure 2C-(a)). In this model, an electron in the *σ*_g_ orbital of H_2_ is excited to the surface and an electron in the surface is excited to the *σ*_u_ orbital of H_2_ with the Coulomb interaction, followed by nuclear-spin flip with the Fermi contact hyperfine interaction between the electron in the *σ*_u_ orbital and the hydrogen nuclei leading to the *o-p* conversion. Consequently, a surface electron is excited to the level above the Fermi level (*E*_F_) by the amount of *E*_rot_, and thus *E*_rot_ is dissipated into metal *electron-hole* pairs. Assuming that the electron transfer probability is independent of its energy, the *o-p* transition probability in this model is proportional to the numbers of electron and hole states available. The calculated transition probabilities are plotted in Figure 2B, showing that the transition probability does not change significantly with *T*_S_, which is inconsistent with the experimental data. Therefore, two combination models based on the *XY* model, namely, *E*_rot_ transfer is shared by both electronic transition and phonon excitation through either one-phonon or two-phonon processes, are proposed (Figure 2C-(b)(c)). It should be mentioned that the electron transition process is essential for nuclear-spin flip via the Fermi contact hyperfine interaction. The results of both combination models in Figure 2B show that the transition probability varies substantially with *T*_S_ in contrast with the result based on the *XY* model. Particularly, the tendency of the combination model of electronic transition and two-phonon process is in good agreement with that of the experimentally determined *o-p* conversion rate. This indicates that the rotational energy of H_2_ transfers into not only electrons but also phonons of surfaces (Ueta and Fukutani, 2023).

Recalling the fact that the vibrational energy is transferred to electrons at metal surfaces, this rotational-energy transfer process might be counterintuitive, because *E*_rot_ is transferred into phonons as well as electrons of surfaces despite a metallic surface. The difference between vibrational- and rotational-energy transfer paths could be ascribed to the energy scale of both degrees of freedom. While the magnitude of the rotational energy of H_2_ ( ~ 10 meV) is much smaller than the vibrational energy such as the CO stretch mode (∼0.26 eV), that is comparable with the magnitude of the substrate phonon energy. The energy transfer path might be determined by the energy-scale matching between the molecular degree of freedom and surfaces as a receiver.

## 4 Concluding remarks

We have expounded a recent advance in the *o-p* conversion study of H_2_, dealing with the fast conversion in the two novel systems characterized by the matrix isolation and molecular chemisorption. It is notable that the conversion time-scale was ~ 10^2^ s or shorter in spite of the non-magnetic properties of these systems. While a variety of metal oxides have been investigated as the magnetic catalysts of the H_2_ *o-p* conversion for many decades, expanding needs for the efficient H_2_ storage and high-performance electrodes for the water electrolysis promotes the studies of H_2_ interacting with non-magnetic substances such as MOFs and carbon nanomaterials. Since the *o-p* conversion involving both the spin- and energy-transfers influences the storage and chemical reaction of H_2_, further studies of the conversion mechanism in the non-magnetic systems are required to reveal the determinant of the conversion time-scale. The anisotropy of a confining potential should be one of the key factors, and the temperature dependence of the conversion rate will provide essential information on the rotational-relaxation pathways also in other systems.
